# Expression of paclitaxel-inactivating CYP3A activity in human colorectal cancer: implications for drug therapy

**DOI:** 10.1038/sj.bjc.6600494

**Published:** 2002-09-04

**Authors:** C Martínez, E García-Martín, R M Pizarro, F J García-Gamito, J A G Agúndez

**Affiliations:** Department of Pharmacology, Medical School, University of Extremadura, Avda. de Elvas s/n, E-06071, Badajoz, Spain; Department of Biochemistry, School of Biological Sciences, University of Extremadura, Avda. de Elvas s/n, E-06071, Badajoz, Spain; Department of Surgery, Medical School, University of Extremadura, Avda. de Elvas s/n, E-06071, Badajoz, Spain

**Keywords:** paclitaxel, metabolism, colorectal, cancer, tissue, resistance

## Abstract

Cytochrome P450 3A is a drug-metabolising enzyme activity due to CYP3A4 and CYP3A5 gene products, that is involved in the inactivation of anticancer drugs. This study analyses the potential of cytochrome P450 3A enzyme in human colorectal cancer to impact anticancer therapy with drugs that are cytochrome P450 3A substrates. Enzyme activity, variability and properties, and the ability to inactivate paclitaxel (taxol) were analysed in human colorectal cancer and healthy colorectal epithelium. Cytochrome P450 3A enzyme activity is present in healthy and tumoral samples, with a nearly 10-fold interindividual variability. Nifedipine oxidation activity±s.d. for colorectal cancer microsomes was 67.8±36.6 pmol min^−1^ mg^−1^. The *K*_m_ of the tumoral enzyme (42±8 μM) is similar to that in healthy colorectal epithelium (36±8 μM) and the human liver enzyme. Colorectal cancer microsomes metabolised the anticancer drug paclitaxel with a mean activity was 3.1±1.2 pmol min^−1^ mg^−1^. The main metabolic pathway is carried out by cytochrome P450 3A, and it is inhibited by the cytochrome P450 3A-specific inhibitor ketoconazole with a *K*_I_ value of 31 nM. This study demonstrates the occurrence of cytochrome P450 3A-dependent metabolism in colorectal cancer tissue. The metabolic activity confers to cancer cells the ability to inactivate cytochrome P450 3A substrates and may modulate tumour sensitivity to anticancer drugs.

*British Journal of Cancer* (2002) **87**, 681–686. doi:10.1038/sj.bjc.6600494 www.bjcancer.com

© 2002 Cancer Research UK

## 

Among the enzyme families that compose the cytochrome P450 multienzymatic system, the most relevant for drug metabolism are CYP1, CYP2 and CYP3. The cytochrome P450 3A (CYP3A) enzyme subfamily represents up to 60% of total cytochrome P450 in human liver samples (Shimada *et al*, 1994), and it is involved in the metabolism of a huge number of procarcinogens, mutagenic agents and clinically used antineoplastic drugs (for a review, see Guenguerich, 1999). Three isozymes compose the CYP3A subfamily in humans. These are CYP3A4 and CYP3A5, with close catalytic functions, and the foetal form CYP3A7 (Gillam *et al*, 1995; Nelson *et al*, 1996) Recently a new CYP3A gene designated as *CYP3A43* has been described (Gellner *et al*, 2001).

CYP3A-mediated drug metabolism is not limited to liver. Evidences for the expression of CYP3A in extrahepatic tissues have been reported, and in particular the expression of CYP3A enzyme activity or immunoreactive protein in diverse segments of intestinal epithelium has been shown. This includes oesophageal squamous mucosa (Hughes *et al*, 1999), duodenum, gallbladder (Yokose *et al*, 1999) and jejunum (Sandstrom *et al*, 1999). The fact that intestinal CYP3A significantly contribute to the ‘first pass’ metabolism of several drugs (Kolars *et al*, 1991) indicates that local metabolism in intestinal epithelium is a relevant factor in individuals treated with CYP3A substrates. Evidences indicating the presence of CYP3A4 and CYP3A5 mRNA transcripts in human colorectal epithelium and in cultured colorectal cancer lines have been reported (Kolars *et al*, 1994; Mckinnon *et al*, 1995; Fontana *et al*, 1999; Yao *et al*, 2000; Nakamura *et al*, 2002). However the presence of functional CYP3A enzyme activity in colorectal epithelium has not been demonstrated to date.

Major clinical implications of the putative presence of CYP3A in colorectal epithelium derive from the use of anticancer drugs that are CYP3A4 substrates in colorectal cancer therapy. CYP3A-mediated drug metabolism may be a relevant clinical factor in tumour sensitivity to these anticancer drugs. If functionally active CYP3A enzyme activity is present in colorectal cancer tissue, the enzyme would permit to cancer cells the metabolism of substrates such as cyclophosphamide or ifosphamide that are activated by CYP3A. By turn, the presence of CYP3A activity would confer to cancer cells the ability to inactivate two widely used taxanes, such as paclitaxel and docetaxel and vinca alkaloids that are CYP3A4 substrates (Guengerich, 1999; Yao *et al*, 2000).

Whether functional CYP3A enzyme activity is present in colorectal tissue with extents of activity high enough to permit intracellular drug inactivation, and many related questions remains to be answered. In this study we investigated the presence of functionally active CYP3A activity in human colorectal cancer tissue. The extent of the enzyme activity, interindividual variability, the properties of the cancer enzyme, and the occurrence of qualitative and quantitative differences on the enzyme activity between healthy and cancer tissues were studied. The role of the CYP3A enzyme as a putative local inactivation mechanism for the anticancer drug paclitaxel in cancer tissue was also analysed.

## METHODS

### Chemicals

Nifedipine and oxidised nifedipine were obtained from Ultrafine Chemicals (Manchester, UK). Ketoconazole was from ICN Biomedical Inc. (OH, USA). NADPH, glucose-6-phosphate and glucose-6-phosphate dehydrogenase were purchased from Boehringer Mannheim (Barcelona, Spain). Paclitaxel was purchased from Sigma Chemical Co. (Madrid, Spain). These and all other chemicals used in this study were of analytical grade. Water was filtered through a Milli Q water system (Millipore Corp., Bedford, MA, USA).

### Patient selection and preparation of samples

Microsomes were prepared from biopsy specimens from 17 patients that underwent surgical resection of colorectal cancer. Both, cancer tissue and healthy surrounding tissue were collected. White Spanish patients (nine males, eight females) with age (s.d. and range) 65.4 (11.1, 45–78) years, diagnosed of colorectal carcinoma were included in the study from January 2000 to January 2001. In every patient the diagnosis of carcinoma was based on the histological data of the surgical resection specimens. Human liver samples were obtained from five unrelated white Spanish patients (three males, two females) with ages (s.d. and range) 47.8 (12.3, 32–61) years, who underwent laparotomy for pathologies not related to liver disease (e.g. cholelitiasis without cholestasis, nonhepatic abdominal tumours). The samples were flash-frozen and stored at −80°C until analysis. Informed consent was obtained from all patients, and all individuals requested agreed to participate in the study. The protocol of the study was approved by the Ethics Committee of the University Hospital Infanta Cristina (Badajoz, Spain). The preparation of microsomal fractions and the measurements of protein concentration were performed as described elsewhere (García-Agúndez *et al*, 1990). All samples were minced with scissors and homogenised at 4°C in a Polytron PTMR2100 (Kinematica AG, Switzerland). Due to differences on toughness among the different tissues analysed, standard homogenisation times were 90 s for liver samples and 180 s for colorectal samples.

### Assay for CYP3A4 enzyme activity

The CYP3A4 activity was studied by using the enzyme-specific substrate nifedipine as described by Guengerich *et al* (1986), except that the incubation volume was reduced to 30 μl and the amount of microsomal protein used was 75 to 150 μg. The reaction was stopped with the addition of 6 μl acetonitrile. The samples were immediately frozen for 10 min and centrifuged for 10 min in a microfuge at maximum speed.

Twenty μl of the supernatant were injected onto an Spherisorb S3 ODS2 4.6×150 mm column (Waters Corporation, Milford, MA, USA) and the elution of nifedipine and its oxidised metabolite was monitored at 254 nm. The flow rate was 1.25 ml min^−1^ and the mobile phase consisted of 0.1% diethylamine, pH 6 and 38% acetonitrile. The oxidised metabolite and nifedipine eluted at 8.0 and 9.0 min, respectively. Once determined that the *K*_m_ values were similar in all tissues studied, a high nifedipine concentration (200 μM) was used to assess the *V*_max_ values for every individual sample. The inhibitory effect of ketoconazole was tested by using identical protein concentration in all tissues studied because ketoconazole is lipophilic, and the unbound concentration of the drug may be influenced by total protein content in the assay mixture.

### Assay for paclitaxel metabolism

Human liver microsomes and colorectal cancer microsomes were assayed for paclitaxel metabolism as described elsewhere (Harris *et al*, 1994) except that the incubation volume was reduced to 30 μl, and the amount of microsomal protein was 75 to 150 μg. The reactions were stopped by the addition of 9 μl acetonitrile. The samples were frozen during 10 min and then centrifuged during 10 min in a microfuge at maximum speed. Twenty μl of the supernatant were analysed for paclitaxel and its metabolites by HPLC (Huizing *et al*, 1995).

All the enzyme activity analyses were performed in amber vials to prevent light degradation of substrates and metabolites. All measurements were done within incubation time and under linear conditions for microsomal protein concentration. Results obtained from incubation mixtures of 30 μl were identical to these from incubation mixtures of 250 μl that were randomly used as controls for reaction volume. All the assays included samples that were stopped at time=0 and blanks without microsomes and/or without substrate. All the experiments were done at least for triplicate. Kinetic data were evaluated according standard procedures by graphical analysis of Lineweaver-Burk, Dixon and Hill's plots. All the results given are mean±s.d. of three or more measurements done under identical conditions.

### Analyses for mutations at the* CYP3A4* gene

Genomic DNA was obtained from all biopsy specimens analysed in this study, by using standard methods (Neitzel, 1986). Two mutations affecting the *CYP3A4* gene were analysed in DNA from cancer and healthy colorectal epithelium for all the samples included in the study. These mutations were selected due to the relatively high frequency of allelic variants containing these mutations among Caucasian individuals, as compared to other mutated gene variants (Sata *et al*, 2000; Cavalli *et al*, 2001; Eiselt *et al*, 2001; Hsieh *et al*, 2001). The allelic variants analysed were CYP3A4*1B and CYP3A4*2. We have shown that among Spaniards CYP3A4*1B is present with an allele frequency over 5% (García-Martín *et al*, 2002). The analyses for CYP3A4*1B were carried out by amplification-restriction procedures (Cavalli *et al*, 2001). The analysis for CYP3A4*2 was carried out by direct sequencing of amplified PCR products as follows: The amplification of the corresponding gene region was carried out as described elsewhere (Sata *et al*, 2000). The sequencing mixture containing 2 μl of the purified PCR products and 60 nM of the corresponding primer, was assembled according the instructions of the manufacturer (dRhodamine terminator cycle sequencing kit, Applied Biosystems). Automated sequencing was carried out in an Abi Prism 310 genetic analyser (Applied Biosystems). The sequencing conditions were as follows: after a loading time of 60 s, the samples were electrophoresed during 120 min at a voltage of 12 kV, with an intensity of 4 μA. The temperature was 50°C.

## RESULTS

The 17 human healthy colorectal epithelium samples analysed showed measurable levels of nifedipine oxidase activity. The mean value±s.d., as measured with 200 μM nifedipine was 97.9± 74.8 pmol min^−1^ mg protein^−1^. The enzyme activity displayed a nearly 10-fold interindividual variability with values ranging 33.1 to 294.8 pmol min^−1^ mg protein^−1^ (95% confidence interval=59.5–136.4). The observed variation in enzyme activity was independent of mutations corresponding to CYP3A4*1B and CYP3A4*2 gene variants, since none of the subjects participating in the study carried any of these mutated genes.

Colorectal cancer microsomes displayed ability to oxidise nifedipine with a mean value±s.d. of 67.8±36.6 pmol min^−1^ mg protein^−1^. A nearly 10-fold variability in the enzyme activity was also observed in colorectal cancer microsomes, with values ranging 17.0 to 156.1 pmol min^−1^ mg protein^−1^ (95% confidence interval=49.0–86.5). Genotyping analyses of the cancer samples fully corresponded to that of healthy tissue for every individual analysed. Therefore mutations corresponding to the gene variants analysed are not responsible for the observed interindividual variability in enzyme activity in cancer tissue.

**Figure 1:**
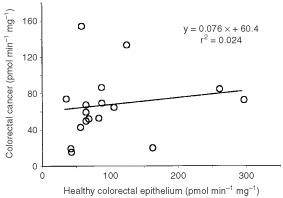
Correlation between CYP3A-nifedipine oxidation activities in healthy colorectal epithelium and colorectal cancer tissue. Linear trend data are shown within the graph.

The mean cancer activity represented 70% of the mean activity in healthy tissue, but a high variability in the differences of the enzyme activity between cancer and healthy tissue was observed. Extreme cases ranged from an 85% decrease of the enzyme activity in cancer tissue to a nearly three-fold increase in the enzyme activity, as compared to healthy tissue. A lack of correlation was observed between the activities of microsomes from colorectal cancer and healthy epithelium (Figure 1

; *r*^2^= 0.024).

**Figure 2:**
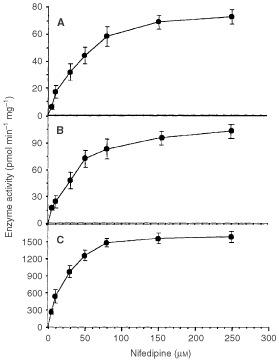
Saturation kinetics for nifedipine in pooled microsomes from different tissues. (**A**) colorectal cancer; (**B**) healthy colorectal epithelium; (**C**) human liver microsomes. Pooled samples were obtained from a mixture of 750 μg of microsomal protein for each of the following samples: 1, 2, 3, 6, 8, 10, 13, 14, 15, 16 and 17. The selection of the samples was based on the availability of tissue (i.e. the biggest surgical specimens were used). Pooled human liver microsomes were obtained from a mixture of 500 μg of microsomal protein each from five white individuals. None of them carried CYP3A4*1B or CYP3A4*2 gene variants. Results are mean±s.d. of at least three independent experiments. The r^2^ values for the double reciprocal plots (not shown) of these data are (**A**) 0.995; (**B**) 0.996; and (**C**) 0.991.

Kinetic analyses of the enzyme activity were performed in pooled microsomal samples from colorectal cancer microsomes (Figure 2A

), healthy colorectal epithelium (Figure 2B) and human liver microsomes (Figure 2C). The *V*_max_ values were smaller in a 20-fold factor in colorectal cancer and in healthy epithelium, as compared with that of human liver microsomes. The *K*_m_ values indicate similar affinity for nifedipine for all tissue enzymes (Table 1

**Table 1:**
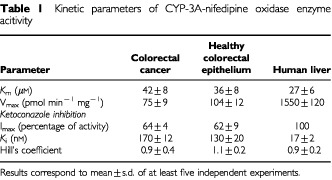
Kinetic parameters of CYP-3A-nifedipine oxidase enzyme acitivity

). The effect of the CYP3A inhibitor ketoconazole added to the reaction mixture was tested in pooled colorectal cancer microsomes, in healthy colorectal epithelium and in human liver microsomes for comparison. Figure 3

**Figure 3:**
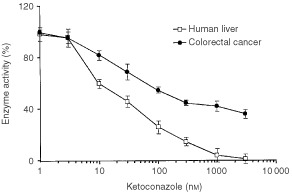
Inhibition of nifedipine oxidase enzyme activity by ketoconazole. The experiments were carried out in pooled microsomes from human liver and colorectal cancer. The percentage of activity is referred to the activity in the absence of inhibitor. Results are mean±s.d. of at least three independent experiments.

shows the inhibition curves for cancer and human liver enzyme activities. Inhibition parameters are summarised in Table 1. It is to be noted that ketoconazole inhibited enzyme activities from all tissues with similar Hill's coefficient values. However, colorectal enzyme activities show decreased sensitivity to the inhibitory effect of ketoconazole, as compared with the human liver microsomal enzyme.

### Paclitaxel metabolism in colorectal cancer

**Figure 4:**
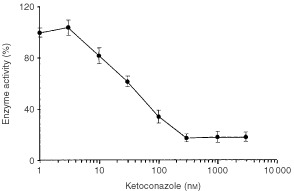
Inhibition of 3′-p-hydroxypaclitaxel formation by ketoconazole in colorectal cancer. The experiments were carried out in pooled microsomes from colorectal cancer. The percentage of activity is referred to the activity in the absence of inhibitor. Results are mean±s.d. of three independent experiments.

Microsomes from five randomly selected colorectal cancer samples and three randomly selected human liver samples were analysed for paclitaxel metabolism. For this, microsomes were incubated with 10 μM paclitaxel in the presence of an NADPH-regenerating system. Paclitaxel was metabolised at a rate of 3.1±1.2 pmol min^−1^ mg protein^−1^ in colorectal cancer samples. The activity observed in human liver microsomes was 37.8±9.6 pmol min^−1^ mg protein^−1^. In order to identify tissue-specific qualitative differences in paclitaxel metabolism, the rate formation of two paclitaxel metabolites was analysed. The predominant metabolite in human liver microsomes, with a mean activity±s.d. of 26.8±12.1 pmol min^−1^ mg^−1^ was 6-alpha-hydroxypaclitaxel. The secondary metabolite 3′-p-hydroxypaclitaxel, a CYP3A4 product (Harris *et al*, 1994), was produced at a rate of 10.9±9.0 pmol min^−1^ mg^−1^. In colorectal cancer microsomes both metabolites were also identified. Interestingly, the predominant metabolite in colorectal cancer is 3′-p-hydroxypaclitaxel with a mean activity of 2.6±0.8 pmol min^−1^ mg^−1^. The rate of formation of 6-alpha-hydroxypaclitaxel in colorectal cancer microsomes was 0.5±0.4 pmol min^−1^ mg^−1^. Figure 4

shows the inhibition curve of 3′-p-hydroxypaclitaxel formation by ketoconazole in colorectal cancer microsomes. Over 80% of the cancer enzyme activity is inhibited with ketoconazole concentrations over 300 nM. The mean±s.d. of the IC_50_ value is 30.6±4.8 nM and the Hill's coefficient was 1.02±0.16.

## DISCUSSION

The development of resistance to the cytotoxic effect of taxanes is related to a decrease in the ability to accumulate the drug within the cells, changes in the stability of microtubules, interference in the ability of the drug to bind tubulin, and changes in the expression of tubulin genes (Cabral, 2001). This study is aimed to assess the potential of CYP3A activity in colorectal cancer as a putative resistance mechanism capable to influence anticancer therapy with drugs that are CYP3A4 substrates. The findings reported in this paper provide evidences for local drug inactivation within cancer cells.

Extrahepatic drug metabolism is a major factor that contributes to clinical drug effects. The most obvious mechanism is intestinal metabolism that often plays a key role in drug inactivation. In addition, tissue-specific expression of drug metabolising enzymes is likely to be involved in local drug inactivation. If cancer develops in a tissue that constitutively expresses an anticancer-inactivating enzyme, it might be important to elucidate whether the cancer cells maintain the ability to express the enzyme activity. In this regard, an association between the expression of the dihydropyrimidine dehydrogenase enzyme and 5-fluorouracil resistance in human cancer cells has been demonstrated (Kirihara *et al*, 1999). Similar findings have been reported for glutathione transferase enzyme activity and resistance to mitomycin C (Singh *et al*, 1992; Nishiyama *et al*, 1997).

To date only indirect data indicated the presence of CYP3A in healthy colorectal tissue. No previous studies on CYP3A activity have been performed in human colorectal cancer. This study provides novel information on several relevant issues, including the demonstration of the presence in colorectal cancer cells of a constitutive enzyme that permits the inactivation of CYP3A4 substrates. The extent and variability of the activity, the kinetic properties, and the implications of the tumour enzyme in paclitaxel metabolism are also analysed. A key factor when investigating local metabolism in cancer cells is to elucidate whether the expression level of enzymes is modified during the carcinogenesis process. This was analysed by comparing the cancer enzyme activity and the activity in surrounding healthy epithelium. Paired Wilcoxon comparison of data shown in Figure 1 revealed non-significant differences between healthy and colorectal tissue activities. However it should be noted that there is an increase in the activity in some cases and a decrease in some others. The rationale for this observation is unknown. Part of the interindividual and intraindividual variability on enzyme activity may be related to sampling or to differences in cellularity of individual specimens. Nevertheless, the regulation of the expression of CYP3A enzyme activities is poorly understood, and it cannot be ruled out that factors affecting gene expression may be modified in some cancer cells during the carcinogenesis process. Minor differences in CYP3A mRNA concentration between human colorectal cancer and healthy tissue have been reported recently (Nakamura *et al*, 2002). This fully agrees with the findings concerning enzyme activity reported in the present study. Differences in kinetic properties and susceptibility to ketoconazole inhibition, exhibited by malignant colonic epithelium versus normal colonic epithelium, are negligible and indicate the absence of substantial qualitative differences of CYP3A activity between normal and malignant tissue.

Both, healthy tissue and colorectal cancer enzymes, show *K*_m_ values for nifedipine that are similar to that of human liver. However it is to be noted that ketoconazole, a CYP3A-specific inhibitor (Guengerich, 1999) does not fully inhibit colorectal enzymes, as it does in liver. This suggests the occurrence of organ-specific differences on CYP3A enzyme activity. These differences may be related to the relative contribution of CYP3A enzymes in colorectal epithelium (Fontana *et al*, 1999), since it has been shown that in microsomal preparations containing CYP3A4 and CYP3A5, an increase in the molar fraction of CYP3A5 drives the apparent *K*_i_ value for ketoconazole to high values (Gibbs *et al*, 1999). The high sensitivity of CYP3A activity to the chemical environment, and in particular to levels of coenzymes such as cytochrome b5 and NADPH reductase (Buters *et al*, 1994; Gillam *et al*, 1995) may also underlie organ-specific differences. In addition, tissue differences in lipid composition may influence the enzyme activity and the inhibitory effect of ketoconazole. Regardless these tissue differences, tumour activity is inhibited to a high extent by submicromolar ketoconazole concentrations, as shown in Figure 4.

The main paclitaxel metabolite in colorectal cancer is 3′-p-hydroxypaclitaxel, a CYP3A4 metabolite (Harris *et al*, 1994). The wide range in the extent of enzyme activity in cancer samples suggest that local metabolism of anticancer drugs in tumour tissue is expected to occur with a high interindividual variability. Nevertheless, it should be pointed out that with both drugs, nifedipine and paclitaxel, cancer cells have a small fraction of the CYP3A activity in the liver. Although colorectal metabolism could play a relevant role in enterohepatic recycling and reabsorption of paclitaxel, the ability of colorectal tissue to influence systemic disposition of paclitaxel is expected to be of secondary importance. The significance of the activity is rather related to the drug molecules that are inactivated within cancer cells. At usual treatment schedules cancer cells are exposed to paclitaxel concentrations within the low micromolar range (Wu *et al*, 2000), which is high enough to consider the cancer enzyme within the frame of pharmacological relevance.

The potential clinical interest of the findings reported in the present study is based on the possibility to inhibit the cancer enzyme, and thereby the local inactivation mechanism. This study shows that the cancer enzyme activity can be inhibited to a high extent by ketoconazole. It is tempting to speculate that individuals suffering from colorectal cancer tumours with high CYP3A activity would benefit from the combined use of taxanes and CYP3A inhibitors. Although from *in vitro* studies it could be expected that CYP3A4 inhibition cause an increased systemic exposure to paclitaxel, *in vivo* studies revealed that the coadministration of paclitaxel and ketoconazole does not change the plasma concentration of paclitaxel and its 6-alpha-hydroxylated metabolite (Jamis-Dow *et al*, 1997). This is an expected finding since in most individuals paclitaxel metabolism in liver is mainly carried out by the CYP2C8 enzyme (Sonnichsen *et al*, 1995; Desai *et al*, 1998), that is not substantially inhibited by ketoconazole. In contrast, our findings indicate that local metabolism in colorectal cancer tissue is mainly CYP3A-dependent, and that CYP2C8 metabolism is of secondary importance. This is in agreement with the low level of expression of CYP2C8 in intestine (Klose *et al*, 1999). These findings taken together suggest that the coadministration of ketoconazole may decrease paclitaxel inactivation in colorectal cancer without significant effects on systemic paclitaxel metabolism. However, the high number of drugs that are CYP3A4 substrates and the fact that cancer patients are often simultaneously treated with several drugs, makes it undesirable to cause a systemic inhibition of CYP3A4 in these patients. Efforts should be made to design methods capable to cause a local inhibition of the tumour CYP3A4 enzyme. It should be mentioned that CYP3A4 is also involved in the metabolism of vinca alkaloids (Yao *et al*, 2000). Therefore the presence of CYP3A4 enzyme activity in colorectal cancer cells may also influence tumour sensitivity to these drugs. Further studies should focus on the presence and kinetic properties of CYP3A4 and other drug-inactivating enzymes in other cancers that are treated with CYP3A4 substrates such as breast, lung and ovary cancers.
